# Stability of miR-126 in Urine and Its Potential as a Biomarker for Renal Endothelial Injury with Diabetic Nephropathy

**DOI:** 10.1155/2014/393109

**Published:** 2014-04-17

**Authors:** Yang Liu, Guangqiang Gao, Chun Yang, Kun Zhou, Baozhong Shen, Hongyan Liang, Xiaofeng Jiang

**Affiliations:** ^1^Department of Clinical Biochemistry Laboratory, The 4th Affiliated Hospital of Harbin Medical University, Harbin 150001, China; ^2^Heilongjiang Province Key Laboratory of Molecular Image, Harbin 150001, China

## Abstract

*Background.* The purpose of the present study was to assess the feasibility of using miR-126 in the urine as a biomarker for diabetic nephropathy. *Methods.* miRNAs were extracted from the urine samples of T2DM patients with diabetic nephropathy (DN; *n* = 92), T2DM without DN (*n* = 86), and 85 healthy volunteers using quantitative reverse transcriptase polymerase chain reaction (real-time polymerase chain reaction) analysis. Stability of urinary miR-126 and factors that affected the stability were assessed. A subgroup analysis was also carried out to compare the urinary miR-126 level in T2DM patients well controlled by the treatment versus those who were not well controlled. *Results.* Urinary miR-126 was stable when the urine samples were kept at room temperature for extended period of time, 4°C, −20°C, and −80°C for up to 12 hours or subjected to 10 freeze-and-thaw cycle. Urinary miR-126 was significantly higher in T2DM patients with DN (5.76 ± 0.33 versus 3.25 ± 0.45 in T2DM patients without DN). Successful treatment significantly reduced urinary miR-126 in T2DM patients with DN to 3.89 ± 0.52 (*P* < 0.05). *Conclusion.* miR-126 in the urine is stable and it could be used as a biomarker of DN and to monitor the treatment response.

## 1. Introduction

MicroRNAs (miRNAs) are small, endogenous noncoding RNAs, 21–25 nucleotides in length [1, 2]. Alteration in serum/plasma levels of miRNAs is closely associated with many diseases, including differentiation, inflammation, allergic reactions, diabetes, and several types of cancer [3–6]. For example, plasma miR-208 has been used as a biomarker of acute myocardial infarction [7, 8]. Plasma miR-122 increases during drug-induced liver injury [9–11]. In addition to their presence in cells/tissues and in plasma, miRNAs are also present in other body fluids, including saliva, urine, tears, amniotic fluid, and breast milk. The type and concentration of these miRNAs in body fluids have also been correlated with disease types and pathological processes and can potentially be used as disease biomarkers to monitor physiological and/or pathological states [12–14].

Diabetic nephropathy (DN) is a microvascular complication of type 2 diabetes mellitus (T2DM) and a major cause of end-stage renal disease (ESRD) [15]. Key pathological features of DN include glomerular basement membrane thickening, mesangial expansion podocyte effacement, and glomerular sclerosis [16]. Clinically, DN is often manifested as microalbuminuria and progressive glomerular dysfunction.

A wide variety of biomarkers, including creatinine, kidney enzymes, cystatin C, and injury molecules, have been developed for early detection of acute kidney injury (AKI) [17]. Recent studies implicated miRNAs in DN. For example, several types of miRNAs are upregulated in the kidneys of diabetic mice [18]. Decreased levels of miR-200c, miR-141, miR-429, and miR-192 have been reported in patients with systemic lupus erythematosus than in healthy controls [19].

miR-126 has whole bunch of effects/functions reported, including endothelial cell biology, cancer, cardiovascular disease, Parkinson, DM [20–23]. Lower plasma miRNA-126 has been associated with a loss of endothelial miR-126 expression in patients with T2DM [24].

In the present study, we examined miR-126 concentration in the urine of T2DM patients with versus without DN and the change of urine miR-126 after treatment in T2DM patients with DN. Prior to the formal experiments, a preliminary stability study was conducted to examine the feasibility of using urine miR-126 as a biomarker.

## 2. Subjects and Methods

### 2.1. Study Participants and Diagnostic Criteria

The current study included 92 T2DM patients with DN, 86 T2DM patients without DN, and 85 healthy volunteers who were matched for age, body mass index (BMI), and gender. Blood pressure was measured by two independent staff members blinded to the study grouping with a standard sphygmomanometer. Hypertension was defined as systolic pressure of 140 mmHg or greater, and/or diastolic pressure of 90 mmHg or greater, or when patients had a clear history of hypertension. Patients with a history of coronary artery disease, cerebrovascular accident, or intermittent claudication were excluded from the study. Hypervolemic subjects were also excluded. An informed consent was obtained from all subjects.

The diagnosis of T2DM was established based on the American Diabetes Association criteria [25], with a disease course of ≥5 years. Diagnosis criteria for DN were established on the basis of overt diabetic retinopathy plus macroalbuminuria at >300 mg/d. Retinopathy is demanded to be exist is to ensure that albuminuria is the outcome of diabetic nephropathy rather than a nondiabetic glomerulopathy. A renal biopsy would be the gold standard to distinguish between diabetic nephropathy and a nondiabetic glomerulopathy. But in diabetic patients, a renal biopsy is almost never taken, and many researches have indicated that retinopathy being present is a good alternative for differentiation between diabetic nephropathy and nondiabetic glomerulopathy in type 2 diabetic patients with albuminuria [26–28]. The study was approved by the Ethics Committee of the 4th Affiliated Hospital of Harbin Medical University.

### 2.2. Sample Collection and Preparation

The second morning urine samples were collected after overnight fasting to measure pH, glucose, blood content, and specific gravity as described previously [29]. Then urine samples (500 *μ*L) were centrifuged briefly to remove cells and debris prior to miRNA extraction, using a mirVana PARIS Kit (Ambion, America). miRNAs were transcribed to cDNAs using RevertAidTM reverse transcriptase and miRNA-specific stem-loop primers (Applied Biosystems, America). Each reaction consisted of 17.5 *μ*L dissolved RNA, 7.5 *μ*L master mix (5 *μ*L 5× Reaction Buffer, 2 *μ*L miRNA-specific stem-loop primers, and 0.5 *μ*L 200 U/*μ*L reverse transcriptase), and 1 *μ*L deoxynucleoside triphosphates. A blank control was used in each experiment to ensure that the PCR products were not contaminated.

### 2.3. Quantitative Reverse Transcriptase Polymerase Chain Reaction (Real-Time Polymerase Chain Reaction) Analysis

Quantitative real-time PCR was used to analyze the amount of miRNA in the urine samples. miR-126 was amplified using LightCycler 480 Probes Master kit (Roche Applied Science, Germany) and examined using TaqManTM MicroRNA hsa-miR-126-specific primers (Applied Biosystems). Real-time PCR was performed on a LightCycler 480 II RT-PCR System (Roche Applied Science, Germany) with the following condition: 94°C for 120 seconds followed by 45 cycles of 94°C for 20 seconds, 56°C for 10 seconds, and 70°C for 20 seconds, using the LightCycler 480 Probes Master kit (Roche Applied Science, Germany). Nontemplate reaction controls produced no detectable signals in any of the experiments. Each urine sample was extracted in triplicate, followed by simultaneous reverse transcription and triplicate analysis through real-time PCR. The microRNA level was quantified using the formula 2^(50-Ct)^. Data are expressed in lg unit.

### 2.4. miRNA Stability

Urine samples were collected from 60 healthy individuals and placed in plain glass tubes. Aliquots from each urine specimen were subject to miRNA extraction immediately or after storage at room temperature, 4°C, −20°C, and −80°C for 3, 6, or 12 hours. Some of the aliquots were also subjected to up to 10 freeze-thaw cycles. Data are expressed as percentage to the control immediately after the extraction (without storage).

### 2.5. Effects of Urinary Albumin on miRNA Detection

Effects of albumin were examined by adding albumin standard solution containing urinary albumin, urinary transferring ferritin, urinary immunoglobulin IgG, urine microglobulin *β*, urine microglobulin *α*, and retinol binding protein to the urine samples to a concentration of the trace protein at 0–140 mg/L.

### 2.6. pH and Urinary miRNA Levels

Twelve urine samples were used for this study. The pH of the samples was adjusted to 4.5, 7.0, and 8.0, respectively, using 5 M HCl or 5 M NaOH. Levels of miR-126 in these aliquots were used to assess the effect of pH on miRNA stability.

All the DN patients received insulin, ACEI/ARE, control of blood pressure, and moderate exercise for about six months as previously described [30–34].

### 2.7. Statistical Analysis

Statistical analysis was performed using SPSS software version 16.0 (SPSS Inc., Chicago, IL, USA). All variables are presented as mean ± SD upon normal distribution and as medians (lower and upper quartiles) otherwise. Gene expression level was log transformed prior to a one-way ANOVA statistical analysis. One-way ANOVA was used to compare levels of gene expression between groups. Paired samples *t*-test was used to compare changes of gene expression before and after the treatment. *P* values below 0.05 were considered statistically significant. All probability tests were two-tailed.

## 3. Results

### 3.1. Detection, Quantification, and Stability of miR-126 in Urine Samples

**Figure 1:**
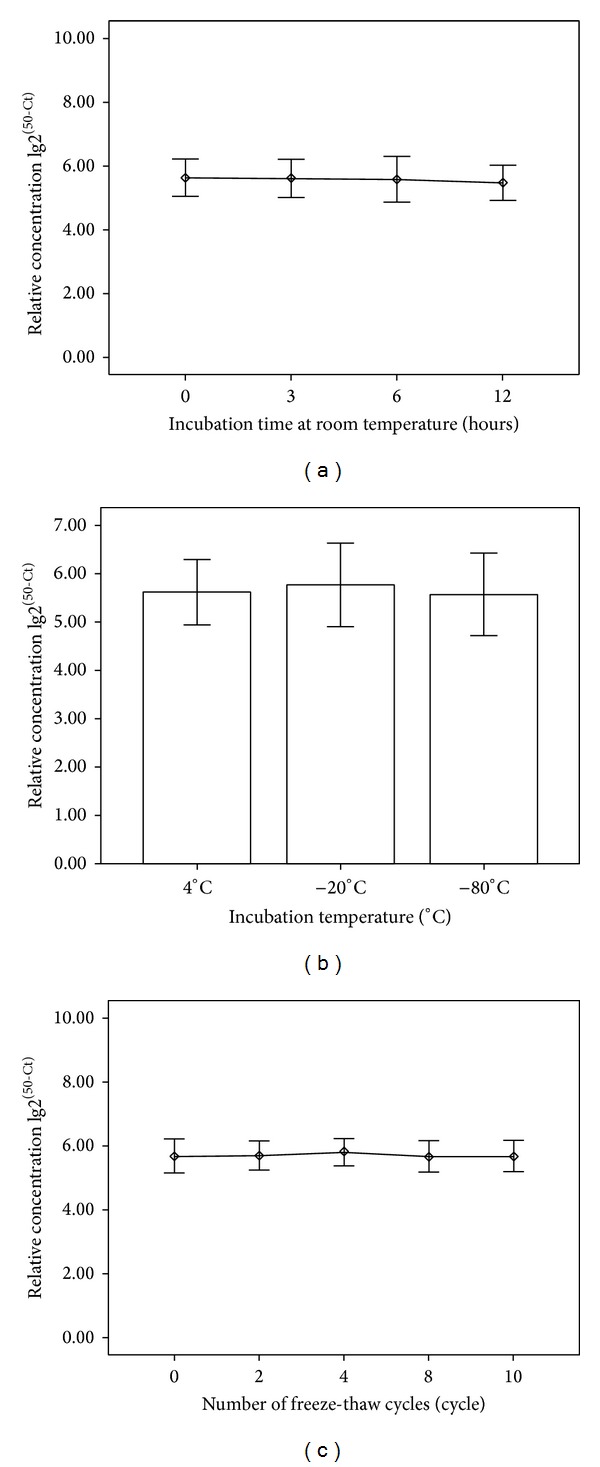
Characterization of miRNA stability in human urine. miRNA levels remain stable when urine is subjected to prolonged room temperature incubation (a), stored at 4°C, −20°C, −80° (b), or freeze-thawed up to multiple times (c). The *P* value is 0.161, 0.134, and 0.643, respectively, *n* = 60.

Using real-time PCR, miR-126 was within detection limit for all tested samples (Figure 1). miR-126 level in the urine did not change significantly after sample storage at room temperature for 3, 6, and 12 hr, respectively (5.65 (5.41–5.76), 5.70 (5.34–5.77), and 5.48 (5.28–5.60) versus 5.65 (5.40–5.79) immediately after extraction; *P* = 0.161; Figure 1(a)). Storage at 4°C, −20°C, and −80°C for 12 hours also did not affect the miR-126 measurement (5.65 (5.40–5.79), 5.72 (5.55–5.98), and 5.61 (5.33–5.89), resp., Figure 1(b)). Freezing and thawing up to 10 cycles prior to miRNA extraction did not affect the miR-126 levels (5.64 (5.58–5.84), 5.76 (5.58–6.00), 5.64 (5.54–5.82), and 5.67 (5.56–5.88) versus 5.68 (5.41–5.74), *P* = 0.643; Figure 1(c)). Sample pH variation (4.5–8.0) did not affect miR-126 level (5.70 (5.60–5.94), 5.67 (5.44–5.78), and 5.64 (5.44–5.96), resp., *P* = 0.635). Urinary miR-126 did not differ with varying albumin concentration (5.73 (5.57–5.87), 5.64 (5.43–5.95), and 5.81 (5.40–5.89), resp., *P* = 0.986).

### 3.2. Elevated Urinary miR-126 in T2DM Patients with DN

**Figure 2:**
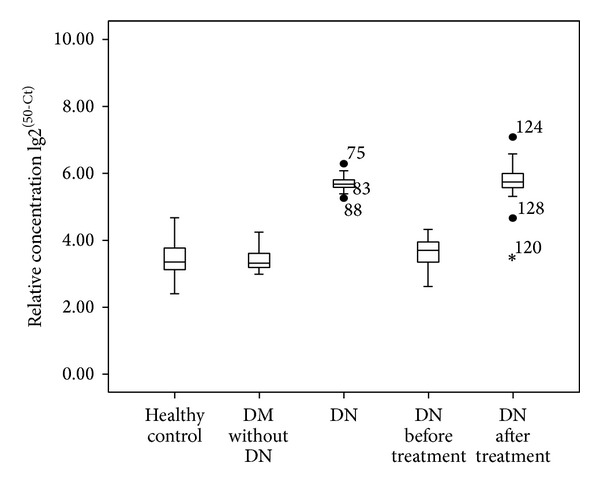
Urinary miR-126 levels of different crowds. Urinary miR-126 level in DN patients was higher than DM without DN patients and healthy controls, *P* = 0.002. However, there was no significant difference between the healthy control and DM without DN, *P* = 0.324. After a period of treatment, urinary miR-126 levels in DN patients were decreased.

The demographic and baseline clinical data of the study subjects are summarized in Table 1. Urinary miR-126 was significantly higher in T2DM patients with DN (5.76 ± 0.33) than that in T2DM patients with no DN (3.76 ± 0.38) or in the healthy subjects (3.25 ± 0.45) (Figure 2 and Table 1, *P* = 0.002). Interestingly, urinary miR-126 level (3.38 ± 0.51) in T2DM/DN patients with a urinary albumin of 2000 mg/L and above was comparable to non-DN (3.25 ± 0.45).

### 3.3. Urinary miR-126 Correlated with Treatment Response in T2DM/DN Patients

Among the 92 patients treated, 80 patients responded to the treatments (patients with decreased urinary albumin level, HbA1c ≤ 7.0%, systolic pressure ≤ 140 mmHg, and diastolic pressure ≤ 90 mmHg) in comparison to the pretreatment baseline (Table 1; (3.89 ± 0.52 effective versus not effective treatment, *n* = 80, *P* < 0.05)). The remaining 12 responded poorly to the treatment, with no significant change of urinary albumin after the treatment (5.76 ± 0.33 before treatment versus 5.24 ± 0.47 after treatment) (Figure 2).

## 4. Discussion

miRNAs in serum and plasma are generally stable [35]. However, the stability of miRNAs in urine, especially during sample collection and preparation, has not been addressed. Our preliminary experiments showed that urinary miRNA (i.e., miR-126) is stable at room temperature, 4°C, −20°C, and −80°C within 12 hours. Also, repeated freezing and thawing up to 10 times did not affect the analysis. The presence of proteins in the urine, up to 140 mg/L, or pH variation (4.5–8.0) did not affect the apparent miRNA level in the urine. Accordingly, urinary miRNA-126 is suitable for use in clinical laboratory setting.

The main experiments in the current study demonstrated higher urinary miR-126 in T2DM patients with DN versus patients without DN. Effective treatment of DN patients significantly reduced the urinary levels of miR-126. These findings indicate that urinary miR-126 could be used as a biomarker for diabetic nephropathy and monitoring the progression and regression of kidney damage in patients with T2DM.

Our findings also suggested a possible role of miR-126 in the pathophysiology of renal damage in T2DM. miR-126 has been found to be highly enriched in endothelial cells and plays a pivotal role in maintaining endothelial homeostasis and vascular integrity [20–22]. Endothelial miR-126 in plasma has been reported to be lower in T2DM patients than in healthy individuals [25]. It is likely that the urine miRNA-126 originates from cells (secreted as exosomes) [36]. Upon leaving the cells, the miRNAs become associated with other molecules and thus protected from degradation. It is likely, as in the case of AlbU, that a RNA-induced silencing complex (RISC) containing miRNA may leak from injured epithelial cells of kidney or glomerular vascular endothelial cells into the urine. This could explain lower urinary miR-126 after effective treatment in the current study.

Also there are many deficiencies in our study. First, there is still no recognized internal reference standard for bodily fluids [37, 38]. We compared log transformation of (50-Ct) values in a given volume of 500 *μ*L urine. The content of urine may be influenced by many factors which probably affect the urinary miRNAs level. Second, our experiments were an indicative research. The sample size was small; we did not study the correlation among the miR-126 level and the stages of DN. And it needs to be seen definitely in the further.

Recently studies show that levels of about 27 miRNAs are significantly upregulated or downregulated in different stages of untreated nephropathy when compared with urinary miRNAs from type 1 diabetes with persistent or intermittent microalbuminuria [39]. Urinary miR-126 may appear to be upregulated or downregulated in the progression of diabetic kidney disease. The early detection of its presence in urine may assist the prediction of the disease course. The threshold of detection of microRNAs by various amplification methods should be increased if we aim to employ miR-126 in urine as a biomarker for determining the severity of diabetic kidney disease and checking the progression of recovery during treatment.

## Figures and Tables

**Table 1:** Clinical characteristics of T2DM patients with and without DN.

Characteristics	Healthy controls (85*)	DM without DN (86)	DN before treatment (92)	DN after treatment (92)		*P*_1_	*P*_2_
				Well controlled (80)	Not well controlled (12)		
Sex (M : F)	36 : 49	47 : 39	57 : 35	50 : 30	7 : 5	0.62	0.75
Age (years ± SD)	52.1 ± 5.6	49.3 ± 4.7	50.3 ± 7.4	51.2 ± 6.3	49.5 ± 8.5	0.72	0.83
BMI Kg/m^2^	24.07 ± 1.21	23.45 ± 1.02	23.17 ± 0.98	24.58 ± 1.16	23.66 ± 1.32	0.68	0.83
SBP (mmHg)	128.4 ± 10.6	129.7 ± 13.2	130.5 ± 11.7	127.8 ± 12.6	129.8 ± 12.9	0.89	0.93
DBP (mmHg)	78.2 ± 8.7	79.1 ± 6.9	80.4 ± 8.5	78.4 ± 7.4	75.3 ± 9.3	0.92	0.87
Fasting glucose (mmol/L)	3.98 ± 0.77	8.37 ± 3.46	5.32 ± 0.25	4.81 ± 2.32	4.54 ± 3.17	<0.05	0.79
HbA1c (%)	5.31 ± 0.32	8.61 ± 0.75	6.26 ± 0.54	6.07 ± 0.61	6.72 ± 0.45	<0.05	0.82
AlbU (g/L)	8.32 ± 3.5	10.32 ± 2.8	437.2 ± 164.6	21.36 ± 8.3	389.7 ± 45.2	<0.05	<0.05
Cys-c (mg/L)	0.62 ± 0.17	0.71 ± 0.21	1.48 ± 1.15	0.67 ± 0.08	1.45 ± 0.78	<0.05	<0.05
UREA (mmol/L)	4.24 ± 1.32	3.98 ± 1.27	6.78 ± 1.90	4.02 ± 1.13	5.79 ± 1.82	<0.05	<0.05
CREA (mmol/L)	53.44 ± 8.26	59.56 ± 7.48	86.43 ± 21.34	54.67 ± 7.14	84.23 ± 19.43	<0.05	<0.05
miR-126 [lg2^(50-Ct)^]	3.25 ± 0.45	3.76 ± 0.38	5.76 ± 0.33	3.89 ± 0.52	5.24 ± 0.47	<0.05	<0.05

*Indicates numbers of subjects in the group. DM: diabetes mellitus; DN: diabetic nephropathy; SBP: systolic blood pressure; DBP: diastolic blood pressure; Hb Alc-A: glycosylated hemoglobin; AlbU: urine albumin; Cys-c: serum cystatin C; CREA: serum creatinine; *P*_1_: comparison to healthy controls, DM without DN, and DN before treatment. *P*_2_: comparison between DN patients well controlled versus not well controlled by the treatment.
